# Elongated styloid process syndrome with prolongation of the superior cornu of the thyroid cartilage: A case report

**DOI:** 10.1016/j.ijscr.2021.106283

**Published:** 2021-08-05

**Authors:** Katsuhisa Sekido, Yasushi Hariya, Kie Yamashiro, Michiko Okita, Masashi Harada, Eiji Nakayama

**Affiliations:** a Department of Dentistry and Oral Surgery, Toyama Red Cross Hospital, 2-1-58, Ushijimahonnmachi, 930-0859 Toyama, Japan; b Department of Oral and Maxillofacial Surgery, and Comprehensive Oral Science, Faculty of Medicine, University of Toyama, 2630 Sugitani, Toyama city, Toyama 930-0194, Japan; c Department of Oral and Maxillofacial Surgery, Teine Keijinkai Hospital, 1-12-1-40, Maeda, Teineku, Sapporo city, Hokkaido 006-8555, Japan; d Division of Oral and Maxillofacial Radiology, Department of Human Biology and Pathophysiology, School of Dentistry, Health Sciences University of Hokkaido, 1757, Kanazawa, Toubetsu, Ishikari, Hokkaido 061-0293, Japan

**Keywords:** Elongated styloid process syndrome, Prolongation of the superior cornu of the thyroid cartilage, styloid process, Styloid resection

## Abstract

**Introduction:**

Elongated styloid process syndrome represents a group of symptoms, such as recurrent throat pain and neck pain, caused by elongation of the styloid process. We report a case of elongated styloid process syndrome with prolongation of the superior cornu of the thyroid cartilage.

**Case presentation:**

A 50-year-old man was referred to our clinic with the chief complaint of discomfort on the right side of his neck. He had no history of any disease. Extraoral findings indicated pain during neck rotation. Computed tomography showed prolongation of the styloid process beyond the mandibular plane and close to the hyoid. Moreover, prolongation of the superior cornu of the thyroid cartilage was detected. The discomfort during rotation of the neck was due to the stimulation by the styloid process. Styloid resection was performed using the extraoral approach under general anesthesia.

**Conclusion:**

Discomfort in the neck was resolved after operation.

## Highlights

• Elongated styloid process syndrome represents a group of symptoms.
• Morphological anomalies or deviations of the thyroid cartilage may cause swallowing discomfort.
• Oral and extraoral methods are used as surgical techniques for styloid hyperplasia.
• There have been no reports of styloid process prolongation with hypertrophic thyroid cartilage.
• There are few reports concerning the prolongation of the superior cornu of the thyroid cartilage.

## 1 Introduction

Elongated styloid process syndrome represents a group of symptoms, such as recurrent throat neck pain, possibly caused by an elongated styloid process that stimulates the cervical nerve [1]. There are many reports on elongated styloid process syndrome treatment, such as corticosteroid administration and surgical removal of the styloid process [2], [3]. However, morphological anomalies or deviations of the thyroid cartilage may also cause swallowing discomfort, and it is necessary to distinguish cervical symptoms [4].

Here, we report a case of elongated styloid process syndrome with prolonged superior cornu of the thyroid cartilage.

## 2 Case report

A 50-year-old man was referred to our department with the chief complaint of discomfort on the right side of his neck. He had no medical history. He had initially become aware of pain on the right side of his neck three years before visiting our department. He first consulted with the oral and maxillofacial department of a local hospital. Computed tomography (CT) at that hospital showed styloid process prolongation; however, the patient reported mild discomfort upon follow-up. The discomfort on the right side of his neck worsened, and he was referred to our hospital for the treatment of his neck symptoms.

**Fig. 1:**
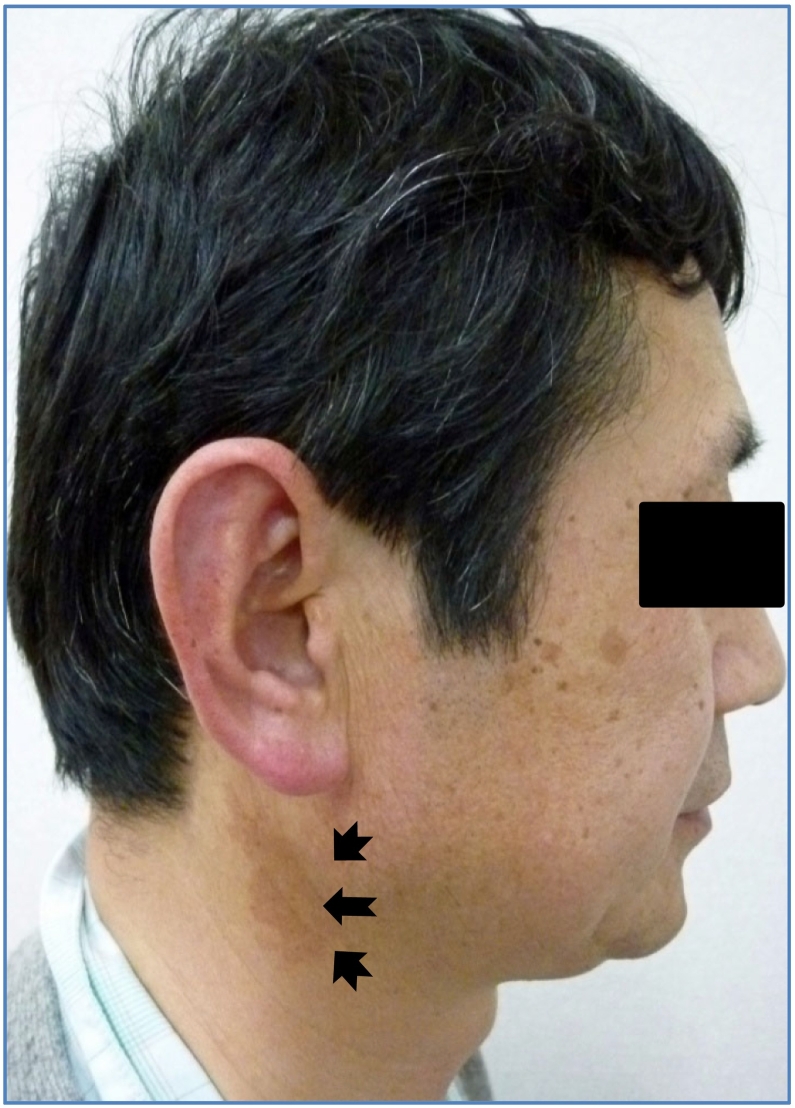
Facial view at the time of initial visit. He felt discomfort during rotation of his neck (arrows).

**Fig. 2:**
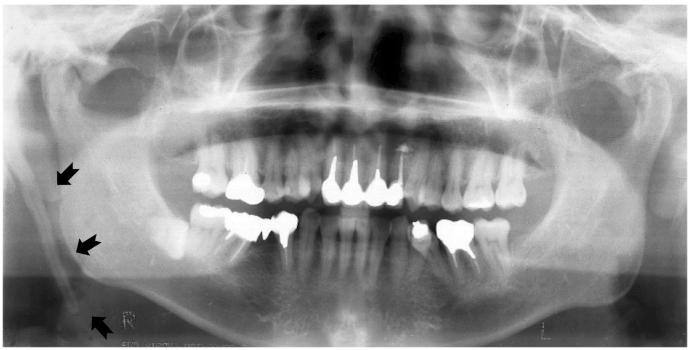
Panoramic radiology of the initial visit. Prolongation of the styloid process beyond the mandibular plane was observed (arrows).

**Fig. 3:**
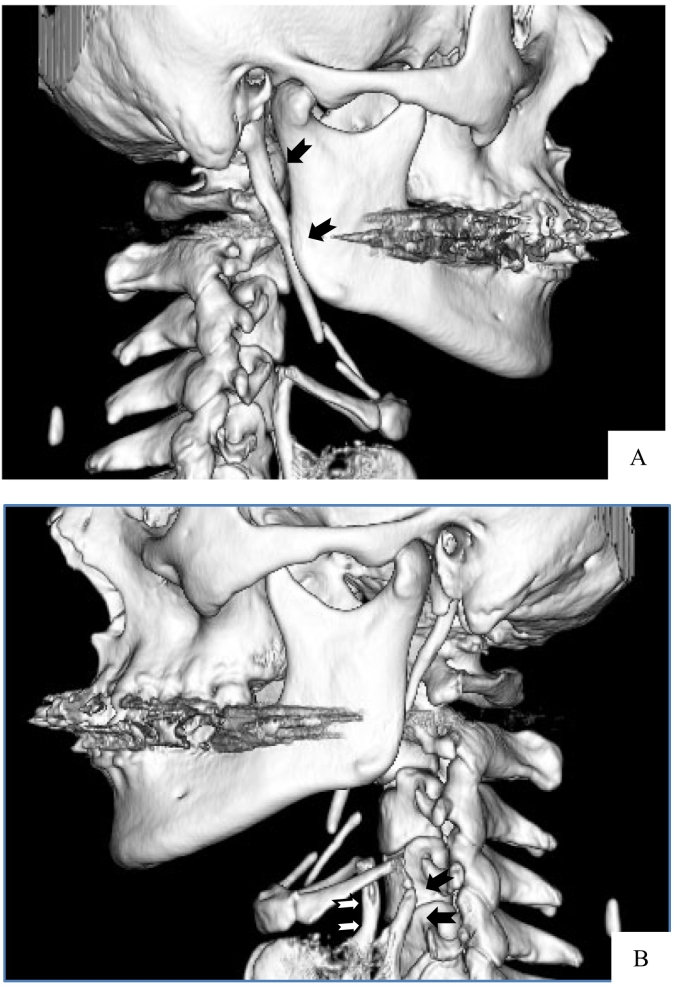
CT image. Prolongation of the styloid process close to the hyoid. Prolongation of the superior cornu of the thyroid cartilage was observed (arrows).

The patient had no systemic disease at the initial visit. He was aware of the discomfort of the right side of his neck when he turned his head (Fig. 1). A rigid bone-like structure was palpable in the right submandibular region. In addition, panoramic radiography revealed an elongated right styloid process beyond the mandibular plane (Fig. 2). CT showed styloid process prolongation on both sides (Fig. 3). The length of the styloid process was 60 mm on the right side. Calcification of the minor horn of the hyoid bone and the styloid process' tip was also observed. Moreover, prolongation of the right superior cornu of the thyroid cartilage was observed, with a length of 23.2 mm. The cervical symptoms might have been due to the extension of the superior thyroid cartilage angle. Still, the discomfort that occurred during the neck rotation coincided with the tip of the styloid process. The patient was diagnosed with symptoms of styloid prolongation [5].

**Fig. 4:**
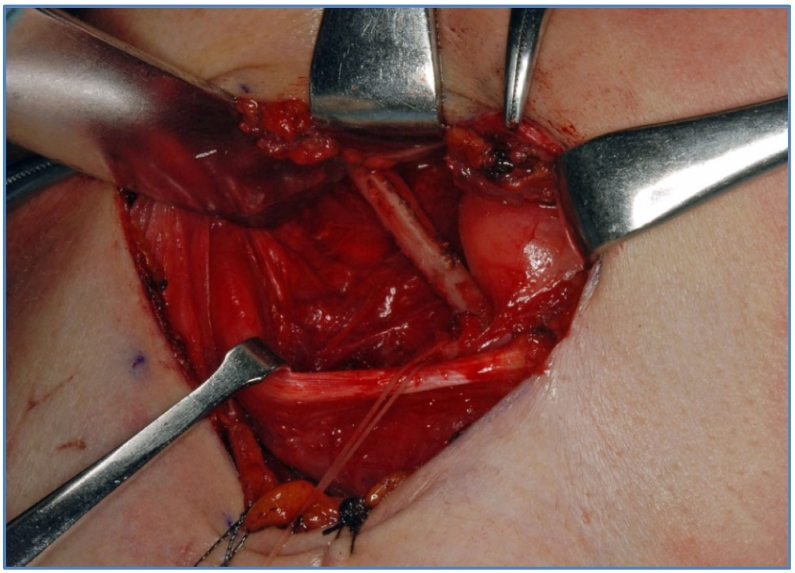
Intraoperative view. The styloid resection was performed by extraoral approach.

**Fig. 5:**
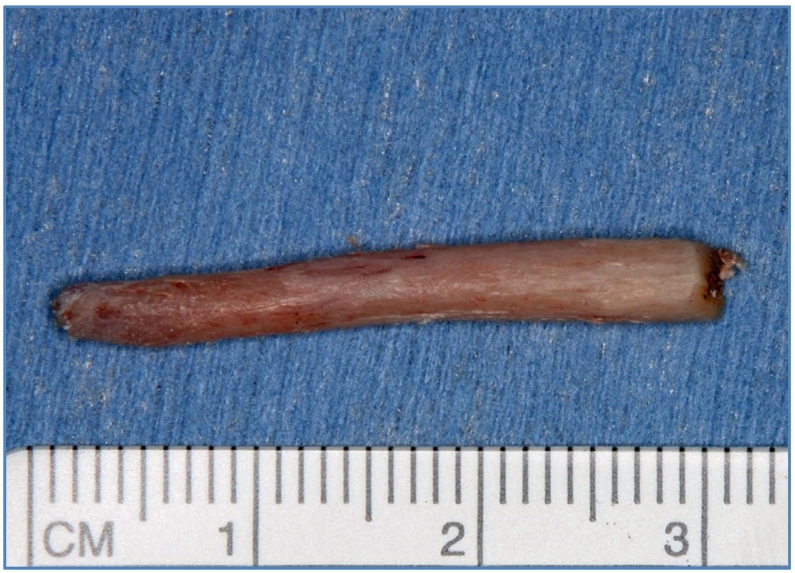
Resected specimen. The specimen shows a 30 × 5 mm large styloid process partly containing a stylohyoid ligament.

Surgical trimming of the elongated styloid process with an extraoral approach was performed under general anesthesia. A skin incision was made in the submandibular region, and detachment was performed to reveal the intermediate tendon of the digastric muscle. A styloid process was observed near the hyoid bone. After the detached surrounding area, the styloid process was resected using an ultrasonic bone scalpel (Fig. 4). The stylohyoid ligament was attached to the tip of the styloid process and, consequently, resected (Fig. 5). The postoperative course was eventful, and the patient's neck discomfort dissipated. Eight years after the operation, the cervical symptoms did not recur.

## 3 Discussion

The styloid process arises embryonically from the Reichert cartilage of the second branchial arch [6]. The syndrome is more frequent in women than in men, and it usually occurs in patients 30 years and older [7], [8]. According to Eagle, 4% of the human population has an elongated styloid process, and only 4% of the above traits present with multiple symptoms [1], [9]. On the other hand, thyroid cartilage originates from the 3rd and 4th arch cartilages. A cartilage rod connecting the hyoid bone body and the thyroid cartilage primordium is formed during the early embryonic period. Subsequently, around three months of fetal life, the cartilage paddle separates, and the cranial side becomes the hyoid bone large angle. The caudal side becomes the superior cornu of the thyroid cartilage. This part is located between the two retracts into the thyrohyoid ligament [10]. There have been many reports regarding malformation of the thyroid bone. However, there are few reports concerning the prolongation of the superior cornu of the thyroid cartilage [11], [12]. The average length of the upper horn of the thyroid cartilage is 14.6 mm from the transition part of the upper horn to the tip of the upper horn [13]. In our case, the length of the right styloid process was 60 mm. The upper horn of the thyroid cartilage was 23.2 mm. Therefore, we diagnosed styloid prolongation and thyroid cartilage upper horn prolongation.

Elongated styloid process syndrome represents a group of symptoms: feeling of a foreign body lodged in the throat, difficulty swallowing, persistent throat pain, pain from turning the head, pain in the infraorbital, infratemporal, ear, and occipital areas, pain opening of the mouth, headache, tinnitus, recurrent pain in the oropharynx, and face otalgia [1]. The causes can be divided into two categories: (1) symptoms caused by physical stimulation of the pharynx, tongue base, and the prolonged hyoid bone, and (2) neural nerve stimulation, such as the glossopharyngeal and cervical sympathetic nerves. The former causes cervical discomfort and swallowing pain, and the latter causes trigeminal neuralgia-like electric shock-like pain [14]. Differential diagnoses include trigeminal neuralgia, glossopharyngeal neuralgia, hyoid bone morphology, thyroid cartilage morphology, atypical facial pain, temporomandibular joint disease, migraine, autonomic imbalance, and anxiety [15]. In reference to morphological abnormalities of the thyroid cartilage, pain and discomfort during swallowing occur due to prolongation and deviation of the superior horn of the thyroid cartilage [10], [11], [12]. In our case, the chief complaint was discomfort of the neck. We needed to differentiate elongated styloid process syndrome from prolongation of the superior horn of the thyroid cartilage. However, our diagnosis was that of an elongated styloid process syndrome because we recognized the tip of the styloid process on palpation and observed the prolongation of the superior horn of the thyroid cartilage. Consequently, dissipation of pain in the neck was observed.

Some reports have explained the mechanisms of styloid process prolongation, such as calcification of the remaining Reichert cartilage, calcification of the stylohyoid ligament, and reactive calcification of the stylohyoid ligament after local stimulation [5], [16]. Moreover, the pattern of styloid process prolongation varies [17]. The patterns were as follows: isolated type, 35%; overlong type, 21.1%; flexion type, 4.5%; partial ossification of ligaments, 9.4%; and protruding type of hyoid bone, 3.0% [17]. On the other hand, prolongation of the superior cornu of the thyroid cartilage is extremely rare, and there are only two reports in our country [11], [12]. One patient experienced dysphagia due to the superior thyroid cartilage protruding into the hypopharynx, which was accidentally discovered during endoscopy. Morphological abnormalities of thyroid cartilage are infrequent. However, they are considered as congenital abnormal cartilage formations, and excessive length of the upper horn is considered an extremely rare condition [11]. In addition to the prolongation of the styloid process, an excessive length of the hyoid bone was also observed; thus, it was considered to be a mixed morphological abnormality. We suspected that the styloid process was prolonged for calcification of the stylohyoid ligament because calcification of both sides of the minor horn of the hyoid bone was detected. There have been no reports of styloid process prolongation with hypertrophic thyroid cartilage to the best of our knowledge, so this case was considered a very rare condition.

Oral and extraoral methods are used as surgical techniques for styloid hyperplasia. Still, in cases where the tip of the styloid process greatly exceeds the lower pole of the almond, it is safe to secure an extraoral approach for better visualization and processing of large blood vessels [14]. For the superior cornu of the thyroid cartilage, thyroidplasties by cervical incision may be recommended when symptoms such as pain during swallowing are substantial [11]. In our case, we performed an extra-oral approach because calcification of both sides of the minor horn of the hyoid bone was observed, leaving the thyroid cartilage. Eight years after the operation, there were no symptoms in his neck. However, careful follow-up is needed because the upper horn of the thyroid cartilage may cause cervical symptoms.

## CRediT authorship contribution statement

KS, YS, KY, MO, and EN contributed to the manuscript preparation. KS, YS, KY, MO, and MH contributed to the patient management. All authors read and approved the final manuscript.

## Declaration of competing interest

The authors declare that they have no competing interests.

## References

[1] Eagle W.W. (1949). Symptomatic elongated styloid process; report of two cases of styloid process-carotid artery syndrome with operation. Arch. Otolaryngol..

[2] Ceylan A., Köybasioglu A., ?elenk F., Yilmaz O., Uslu S. (2008). Surgical treatment of elongated styloid process: experience of 61 cases. Skull Base..

[3] Alamgir A., Baig M.M., Ishfaq U. (2020). Eagle’s syndrome: presentation, diagnosis, and management [presentation]. Med. Coll..

[4] Browning S.T., Whittet H.B. (2000). A new and clinically symptomatic variant of thyroid cartilage anatomy. Clin. Anat..

[5] Agha R.A., Franchi T., Sohrabi C., Mathew G., for the SCARE Group (2020). The SCARE 2020 guideline: updating consensus Surgical CAse REport (SCARE) guidelines. Int. J. Surg..

[6] Kumar K.A., Mowar A., Gupta R., Gulati D. (2014). Eagle’s syndrome- a case report. Int. J. Life Sci..

[7] Bafaqeeh S.A. (2000). Eagle syndrome: classic and carotid artery types. J. Otolaryngol..

[8] Shakeel M., Khan I., Kumar G., Sharma R. (2016). EAGLE’S syndrome: our current experience of the disease and literature review. Jemds..

[9] Balcioglu H.A., Kilic C., Akyol M., Ozan H., Kokten G. (2009). Length of the styloid process and anatomical implications for Eagle’s syndrome. Folia Morphol. (Warsz).

[10] Ilankovan V. (1987). An anomaly of the thyro-hyoid articulation. J. Laryngol. Otol..

[11] Avrahami E., Harel M., Englender M. (1994). CT evaluation of displaced superior cornu of ossified thyroid cartilage. Clin. Radiol..

[12] Counter R.T. (1980). A superior thyroid cornu anomaly: a report of a case-. J. Laryngol. Otol..

[13] Masuyama K. (1959). Racial-anatomical studies on the larynx in Kyushu Japanese. Nippon Jibiinkoka Gakkai Kaiho.

[14] Balbuena L., Hayes D., Ramirez S.G., Johnson R. (1997). Eagle’s syndrome (elongated styloid process). South. Med. J..

[15] Badhey A., Jategaonkar A., Anglin Kovacs A.J., Kadakia S., De Deyn P.P., Ducic Y. (2017). Eagle syndrome: a comprehensive review. Clin. Neurol. Neurosurg..

[16] Murtagh R.D., Caracciolo J.T., Fernandez G. (2001). CT findings associated with eagle syndrome. AJNR Am. J. Neuroradiol..

[17] Monsour P.A., Young W.G. (1986). Variability of the styloid process and stylohyoid ligament in panoramic radiographs. Oral Surg. Oral Med. Oral Pathol..

